# High TGF-β1 expression predicts poor disease prognosis in hepatocellular carcinoma patients

**DOI:** 10.18632/oncotarget.16166

**Published:** 2017-03-13

**Authors:** Li Peng, Xiao-Qing Yuan, Chao-Yang Zhang, Fei Ye, Hui-Fang Zhou, Wen-Ling Li, Zhao-Yang Liu, Ya-Qin Zhang, Xi Pan, Guan-Cheng Li

**Affiliations:** ^1^ Key Laboratory of Carcinogenesis of the Chinese Ministry of Health, Xiangya Hospital, Central South University, Changsha 410078, P.R. China; ^2^ Key Laboratory of Carcinogenesis and Cancer Invasion of Chinese Ministry of Education, Xiangya Hospital, Central South University, Changsha 410078, P.R. China; ^3^ Cancer Research Institute, Central South University, Changsha 410078, P.R. China; ^4^ Department of Clinical Pharmacology, Xiangya Hospital, Central South University, Changsha 410008, P.R. China; ^5^ Institute of Clinical Pharmacology, Central South University, Hunan Key Laboratory of Pharmacogenetics, Changsha 410078, P.R. China; ^6^ Department of Cardiology, the Third Xiangya Hospital, Central South University, Changsha 410100, P.R. China; ^7^ Department of Physiology, Changsha Health Vocational College, Changsha 410100, P.R. China; ^8^ Department of Oncology, the third Xiangya Hospital, Central South University, Changsha 410013, P.R. China

**Keywords:** transforming growth factor beta 1, hepatocellular carcinoma, prognosis, overall survival, meta-analysis

## Abstract

Transforming growth factor beta (TGF-β) promotes the pathogenesis of hepatocellular carcinoma (HCC). We evaluated the associations between TGF-β1 expression and clinicopathological parameters in HCC patients from The Cancer Genome Atlas (TCGA), as well as the prognostic power of TGF-β1 expression. Eligible studies were retrieved from several databases, and effects (hazard ratios (HRs) with 95% confidence intervals (CIs)) for overall survival (OS), disease-free survival (DFS), recurrence-free survival (RFS), metastasis-free survival (MFS), and progression-free survival (PFS) were pooled to assess the prognostic ability of TGF-β1 expression in HCC patients. Twelve qualified articles and our TCGA data comprising 2,021 HCC patients were incorporated. In the TCGA analysis, HCC patients with higher TGF-β1 expression presented a shorter OS than those with lower TGF-β1 expression (HR = 1.42, *p* < 0.05). In the meta-analysis, univariate analyses showed that HCC patients with higher TGF-β1 expression had a shorter OS (pooling HR = 1.71, *p* < 0.01) and DFS/RFS/MFS/PFS (pooling HR = 1.60, *p* < 0.01) than those with lower TGF-β1 expression. In conclusion, our results suggested that high TGF-β1 expression promotes a poor prognosis in HCC patients.

## INTRODUCTION

Hepatocellular carcinoma (HCC) is a common neoplasm of the estimated 782,000 new cancer cases worldwide (50% in China alone), as well as the third leading cause of death from cancer worldwide with nearly 745,000 deaths in 2012 [1, 2]. Despite the growing prevalence in liver cancer, there is a lack of therapies. Besides physical methods (such as radiation, transplant, and operation), there exists just one authorized treatment, which had been followed by a series of costly defeats and controversial candidate drugs [3]. Meanwhile, liver cancer is fatal in both developed and developing countries, with the 5-year overall survival rate generally lower than 20 % [4], and the ratio of mortality to morbidity is 0.95 [1]. HCC is a devastating disease with disappointing outcomes, so it is a very important and urgent task to discover survival outcome predictors, and to furnish potential targets for personalized therapy.

Since its discovery in the early 1980s, transforming growth factor beta (TGF-β) signaling has been increasingly recognized as a cancer promoter [5, 6]. TGF-β orchestrates a favorable microenvironment for cancer cell growth and progression of the epithelial - mesenchymal transition (EMT) [6] and promotes fibrogenesis [7, 8], suggesting TGF-β stimulates HCC pathogenesis and metastasis. TGF-β signaling inhibitors could hinder HCC cell growth and progression by inhibiting the EMT process in distinct experiment models, leading to the clinical investigation of the TGF-β inhibitor LY2157299 (phase II clinical trial, Identifier: NCT01246986,http://clinicaltrials.gov) [6, 9]. However, whether TGF-β1 expression has the potential to predict HCC prognosis is inconsistent. For example, some articles reported that HCC patients with high TGF-β1 expression showed shorter OS and DFS/PFS [10–12], while some other studies revealed negative findings on OS [13, 14]. There is a need to implement meta-analysis and larger sample evaluation, aiming to systematically elaborate on the prognostic power of TGF-β1 expression in HCC patients.

In the current study, we extracted a dataset on HCC from The Cancer Genome Atlas (TCGA), analyzed the associations of TGF-β1 expression with clinicopathological parameters, and evaluated the prognostic power of TGF-β1 expression in HCC patients. In combination with our TCGA analysis, we pooled the prognostic ability of TGF-β1 expression in HCC according to 12 available articles.

## RESULTS

### HCC patient characteristics from TCGA database

One TCGA dataset of liver hepatocellular carcinoma (*n* = 423) was analyzed in July 2016, which was composed of 271 males and 136 females. Moreover, patient characteristics including BMI’ (< 12.23, > 12.23), gender (female, male), age (≤ 62, > 62), weight (≤ 35 kg, > 35 kg), height (≤ 1.68 m, > 1.68 m), clinical stage (I, II, III, IV), etc. were grouped by median and shown in Supplementary Table 1. BMI was grouped by the international obesity standard (< 25, ≥ 25). In addition, HCC patients were stratified by median into two groups of TGF-β1 expression: high and low. There were no clinicopathologic differences between the two groups (*p* > 0.05; Supplementary Table 1).

### High TGF-β1 expression predicted a poor prognosis in HCC patients from TCGA

#### OS

**Figure 1:**
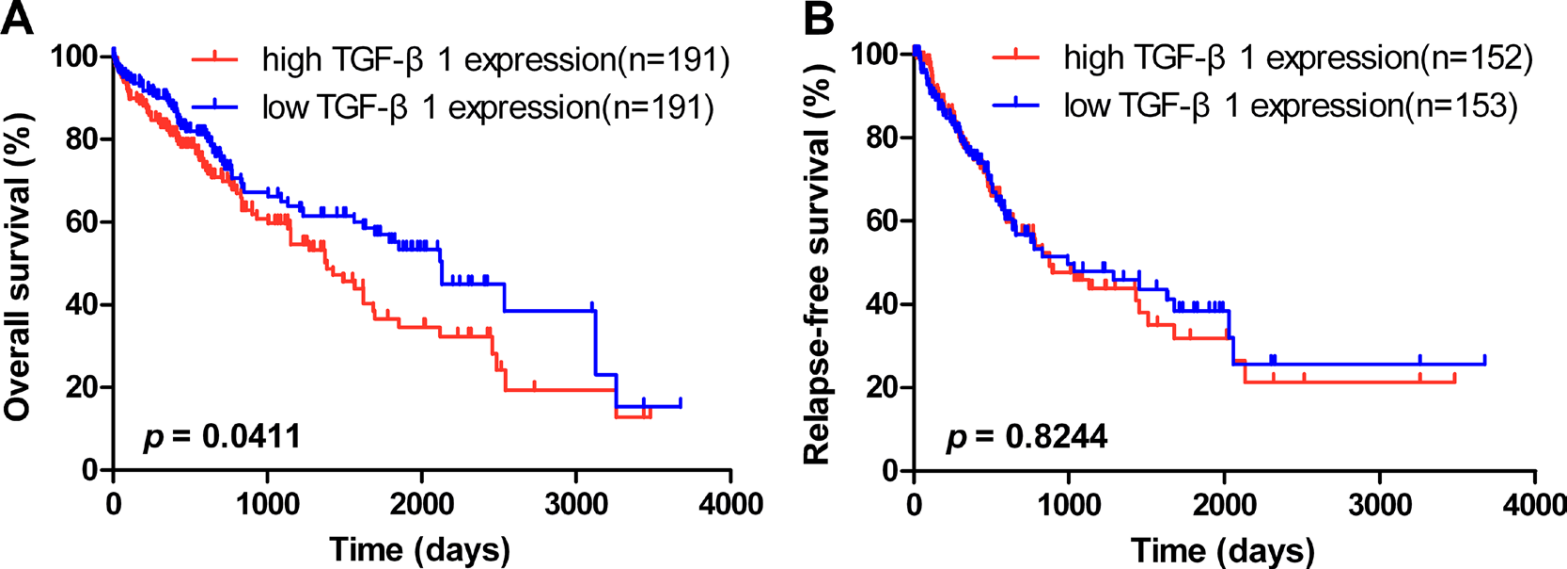
OS and RFS of TGF-β1 expression in HCC patients The overall survival [OS] (**A**) and relapse-free survival [RFS] (**B**) of TGF-β1 expression were shown in hepatocellular carcinoma from TCGA liver hepatocellular carcinoma (LIHC) dataset (LIHC - IlluminaHiSeq; n _OS_ = 382 and n _RFS_ = 305). The median length of OS in high TGF-β1 expression (n _high_ = 191) and low TGF-β1 expression (n _low_ = 191) was 1386 days and 2131 days, respectively (*p* < 0.05).

To further explore the association between TGF-β1 expression and HCC patients’ clinical outcome, the TCGA liver hepatocellular carcinoma cohort was analyzed. HCC patients with higher TGF-β1 expression had a shorter OS in comparison with those with lower TGF-β1 expression (Figure 1A; HR = 1.417, 95% CI = 1.014–1.979, *p* = 0.0411). In addition, subsequent univariate and multivariate COX proportional hazards regression models were conducted to determine the independence of the prognostic power of TGF-β1 expression in HCC patients’ OS. In the COX univariate regression models, higher TGF-β1 expression was correlated with shorter OS (HR = 1.412, 95% CI = 1.011–1.973, *p* = 0.043) in HCC patients. Also, advanced clinical stage (HR = 1.842, 95% CI =1.265–2.681, *p* =0.001) was correlated with shorter OS in HCC patients (Table 1). Multivariate COX regression analysis revealed that a shorter OS trend was seen in HCC patients with higher TGF-β1 expression than those with lower TGF-β1 expression (Table 1; HR = 1.390, 95% CI = 0.972–1.987, *p* = 0.071).

**Table 1:** Univariate and multivariate analysis of clinic pathologic factors for overall survival of 423 HCC patients from TCGA

Risk factors	Univariate analysis			Multivariate analysis		
	HR	95 % CI	*p*	HR	95 % CI	*p*
TGF-β1 expression (high vs. low)	1.412	1.011–1.973	0.043	1.390	0.972–1.987	0.071
Gender (male vs. female)	0.907	0.645–1.277	0.576			
Age (> 62 vs. ≤ 62)	1.333	0.948–1.873	0.096			
Weight (> 35 vs. ≤ 35 kg)	1.372	0.960–1.961	0.083			
Height (> 1.68 vs. ≤ 168 cm)	1.160	0.799–1.686	0.435			
BMI (≥ 25 vs. < 25)	0.049	0.000–392.299	0.510			
BMI’ (> vs. ≤ 12.22363946)	1.011	0.697–1.465	0.955			
Clinical stage (III-IV vs. I-II)	1.842	1.265–2.681	0.001	1.841	1.265–2.679	0.001
Grade (G3–4 vs. G1–2)	1.193	0.839–1.698	0.326			
platelet (> 212 vs. ≤ 212)	1.428	0.992–2.057	0.056			
Albumin (> 4 vs. ≤ 4)	0.734	0.506–1.064	0.102			
Alpha fetoprotein (> 16 vs. ≤ 16)	1.438	0.958–2.157	0.080			
Serum creatinine (> 0.9 vs. ≤ 0.9)	0.962	0.668–1.384	0.833			
Prothrombin time (> 1.1 vs. ≤ 1.1)	1.360	0.942–1.962	0.100			
Total bilirubn (> 0.7 vs. ≤ 0.7)	1.124	0.770–1.640	0.544			
Liver fibrosis ishak score category (fibrosis vs. no fibrosis)	7342.525	0.000–4.062E60	0.894			
Vascular tumor cell invasion (positive vs. negative)	1.287	0.880–1.882	0.193			

#### RFS

We also analyzed the association between TGF-β1 expression and HCC patients’ RFS from the TCGA. There was no difference on RFS between the two TGF-β1 expression groups (Figure 1B; HR = 1.041, 95% CI = 0.7278–1.490, *p* = 0.8244). Subsequent univariate and multivariate COX proportional hazards regression models were conducted to determine the independence of the prognostic power of TGF-β1 in HCC patients’ RFS. In the univariate COX regression models, more advanced clinical stage (HR = 2.332, 95% CI =1.547–3.516, *p* < 0.0001) and higher platelet levels (HR = 1.459, 95% CI = 1.001–2.127, *p* = 0.049) were correlated with shorter RFS in HCC patients (Supplementary Table 2). However, high TGF-β1 expression was not correlated with RFS (HR = 1.041, 95% CI = 0.729–1.488, *p* = 0.825). COX multivariate regression revealed that more advanced clinical stage (HR = 2.769, 95% CI = 1.752 – 4.376, *p* < 0.0001) could predict a worse prognosis on RFS of HCC patients (Supplementary Table 2). To summarize, these TCGA results show that high TGF-β1 expression could act as a prognostic indicator on OS but not RFS in HCC patients.

### Search results for meta-analysis

**Figure 2:**
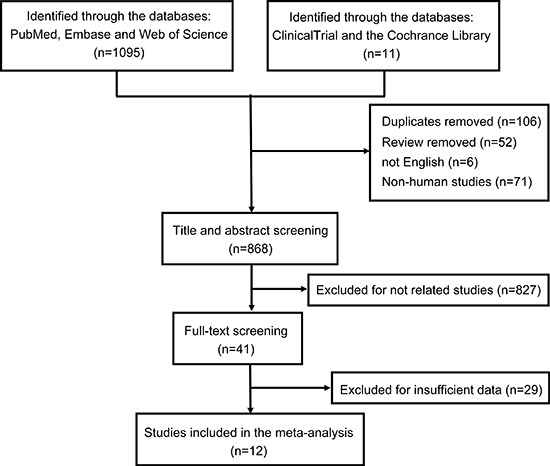
Flow chart of the literature search procedure

A total of 1,106 papers were identified by the systematic literature search, where 106 duplicates, 52 reviews, 6 non-English, and 71 non-human studies were excluded and removed, resulting in 868 papers. Further, 856 articles were removed due to relevance, design, and outcome data through the title, abstract, and full-text screening (Figure 2). Ultimately, 12 papers were incorporated into the meta-analysis.

### Characteristics of eligible studies

Twelve articles [10–14, 24–30] and our TCGA analysis containing a total of 2,021 individuals were incorporated in the meta-analysis. One of the articles contained two datasets [30], which counted as two studies in our meta-analysis. The principal features of these subjects with TGF-β1 expression were displayed in Table 2. Sample size of the published studies ranged from 67 to 350 patients. Interestingly, all included studies were performed in Asia: ten publications originated from China, one from Japan, and one from Singapore. For specimens, two articles were from urine, three from plasma/serum, and seven from tumor tissue. Additionally, the detection method of TGF-β1 expression included in one by quantitative real-time polymerase chain reaction (qPCR), two by 125I radioimmunoassay kit, three by enzyme linked immunosorbent assay kit (ELISA), and six by immunohistochemistry (IHC). Risk of bias (see quality assessment in methods) was appraised basing on the three categories of NOS, and all 12 articles (100%) were regarded as high quality (7–9 stars) (Table 2).

**Table 2:** Characteristics of the eligible studies in meta-analysis

First author	Year	Region	Age	No of patients	Sex (M/F)	Cancer type	Sample type	Tumor stage	Detection method	Survival analysis	Outcomes	Follow-up, months	NOS	Reference
JF Tsai	1997.5	China	55(43–67)	140	111/29	HCC	urinary	I–IV	RIA	Univariate	OS	—	7	[25]
JF Tsai	1997.8	China	58(29–72)	94	76/18	HCC	urinary	I–IV	RIA	Univariate	OS	—	7	[29]
K Okumoto	2004	Japan	65(48–76)	70	55/15	HCC	plasma	I–IV	ELISA	Univariate and Multivariate	OS	4 (0.5–50)	8	[26]
ZL Xiang	2011	China	51	350	300/50	HCC	Tissue	I–III	IHC	Univariate and Multivariate	MFS	53.9 (3.0–120.6)	9	[24]
Y Chao	2013	China	—	73	67/6	HCC	serum	I–III	ELISA	Univariate	OS	—	7	[13]
ZX Chen	2013	China	55.8(29–80)	126	92/34	HCC	tissue	I–IV	IHC	Univariate and Multivariate	OS	—	9	[28]
XH Gai	2014	China	—	96	57/39	HCC	tissue	I–IV	IHC	Univariate and Multivariate	OS	18	8	[27]
C Turato	2014	Singapore	65(41–84)	67	50/17	HCC	tissue	—	qRT-PCR	Univariate	RFS	—	8	[14]
F Ji	2015	China	48(23–75)	84	68/16	HCC	tissue	I–IV	IHC	Univariate and Multivariate	OS; DFS	39 (3–81)	8	[12]
ZH Lin	2015	China	56.9 (23.6–83.1)	91	82/9	HCC	serum	—	ELISA	Univariate and Multivariate	OS; PFS	—	7	[10]
Y Wang	2016	China	—	105	—	HCC	tissue	—	IHC	Univariate	OS; DFS	—	7	[11]
HY Ruan	2016	China	—	T: 184; V: 118	T: 158/26; V: 107/11	HCC	tissue	I–IV	IHC	Univariate	OS; RFS	—	7	[30]

—, there is no corresponding data presented.

DFS, disease-free survival; ELISA, enzyme linked immunosorbent assay kit; HCC, Hepatocellular Carcinoma; IHC, immunohistochemistry; M/F, Male/Female; qRT-PCR, quantitative real-time polymerase chain reaction; RFS, relapse-free survival; RIA, ^125^ I radioimmunoassay kit; OS, overall survival; MFS, metastasis-free survival; PFS, progression-free survival; NOS, Newcastle-Ottawa Scale.

*T*, Test; V, Validation.

### A meta-analysis of prognostic power of TGF-β1 expression in HCC patients

#### OS

**Figure 3:**
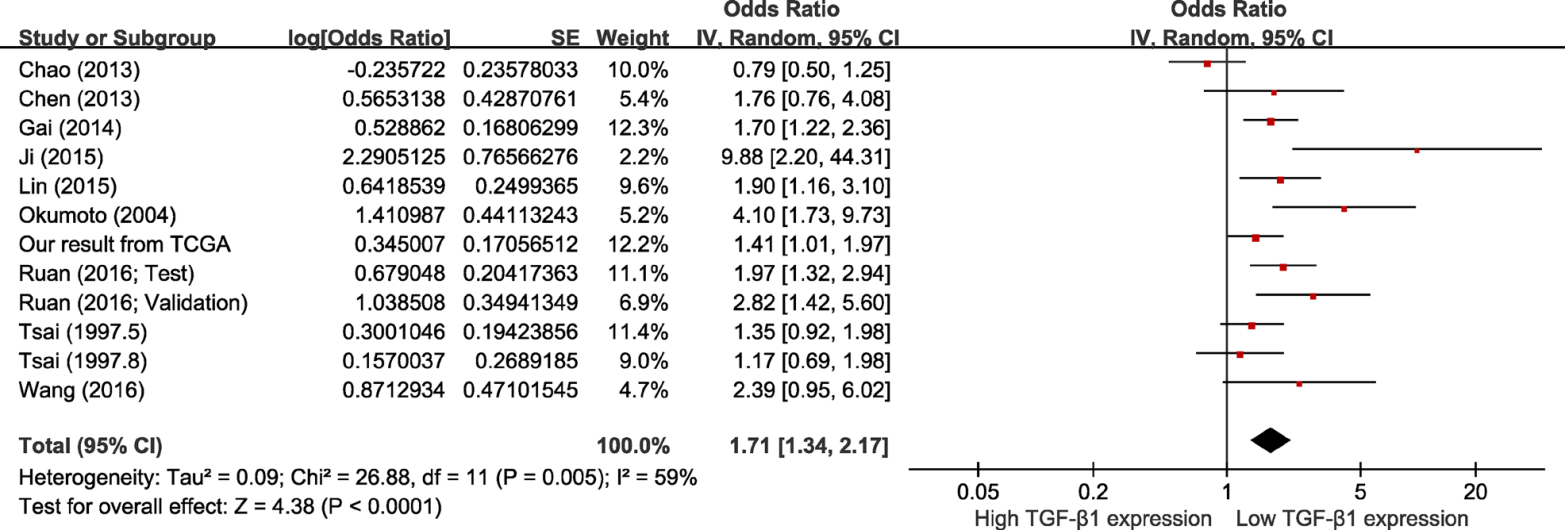
Meta-analysis of the HRs with 95% CI for OS from univariate analysis in HCC The size of the blocks or diamonds represents the weight for the random-effect model in the meta-analysis. HR > 1 indicates that high TGF-β1 expression is correlated with a more unfavorable overall survival (OS).

**Figure 4:**
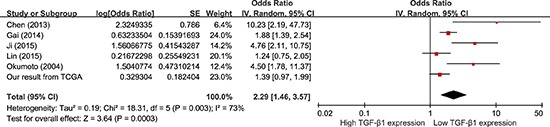
Meta-analysis of the HRs with 95% CI for OS from multivariate analysis in HCC The size of the blocks or diamonds represents the weight for the random-effect model in the meta-analysis. HR > 1 indicates that high TGF-β1 expression is correlated with a more unfavorable overall survival (OS).

Data were derived from COX univariate analysis of all 12 studies, totaling 1604 HCC patients (Figure 3). With a random-effect model, shorter OS was seen with higher TGF-β1 expression in comparison with those with lower TGF-β1 expression (Figure 3; pooling HR = 1.71, 95% CI = 1.34–2.17, *p* < 0.0001). Data were derived from COX multivariate analysis of six studies, totaling 890 HCC patients. With a random-effect model, HCC patients with high TGF-β1 expression presented a shorter OS than those with low TGF-β1 expression (Figure 4; pooling HR = 2.29, 95% CI = 1.46–3.57, *p* = 0.0003). This suggests that high TGF-β1 expression could predict inferior clinical outcomes on OS in HCC patients.

#### DFS/RFS/PFS

**Figure 5:**
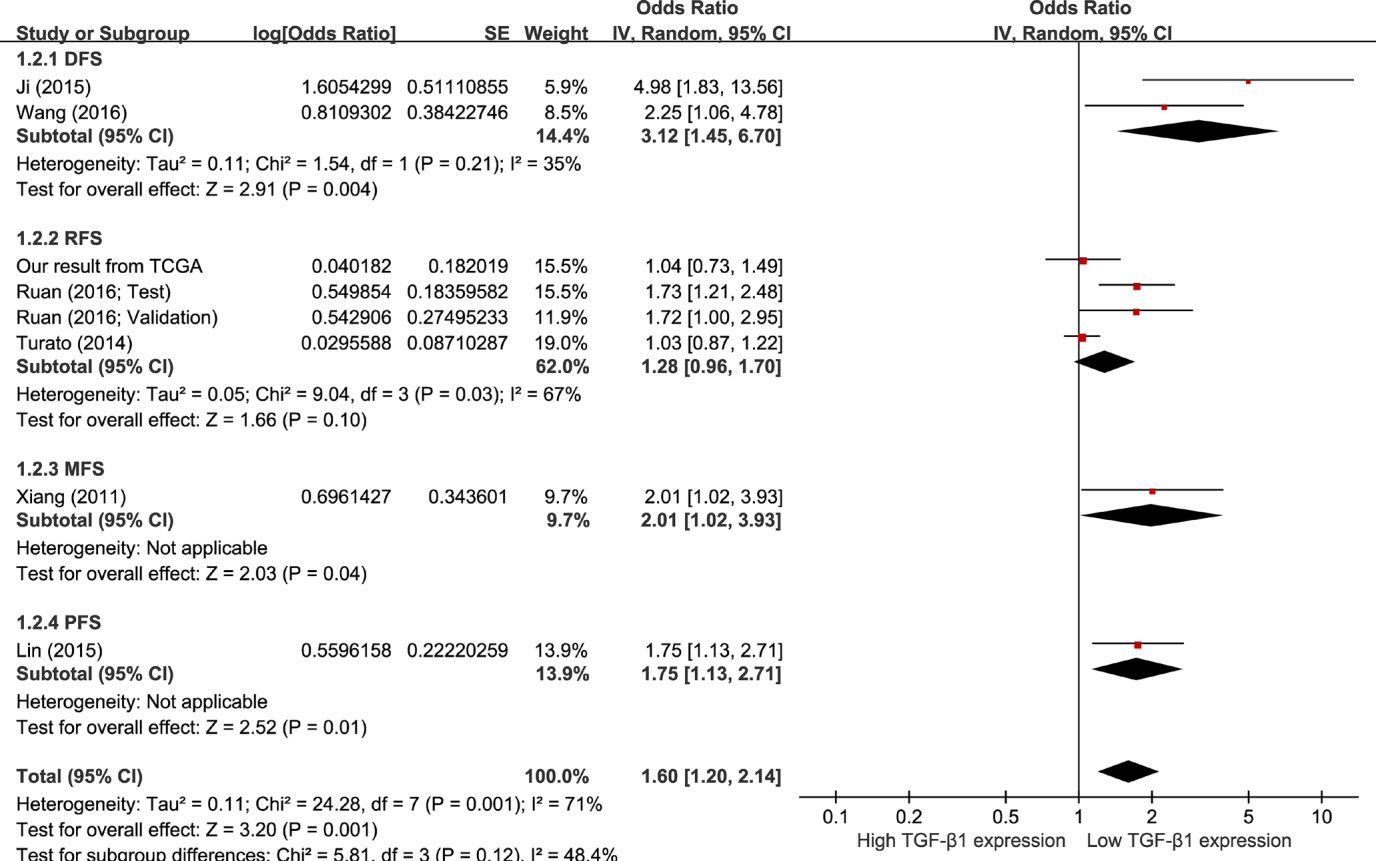
Meta-analysis of the HRs with 95% CI for DFS/RFS/MFS/PFS from univariate analysis in HCC The size of the blocks or diamonds represents the weight for the random-effect model in the meta-analysis. HR > 1 indicates that high TGF-β1 expression is correlated with a more unfavorable disease-free survival (DFS), relapse-free survival (RFS), metastasis-free survival (MFS), and progression-free survival (PFS).

Data were extracted from COX univariate analysis of eight studies, totaling 1,422 HCC patients. With a random-effect model, a shorter DFS/RFS/MFS/PFS was seen in HCC patients with higher TGF-β1 expression in comparison to those with lower TGF-β1 expression (Figure 5; pooling HR = 1.60, 95 % CI = 1.20–2.14, *p* = 0.001). Data were derived from COX multivariate analysis of three studies, totaling 525 patients. With a random-effect model, there was no difference of DFS/MFS/PFS between the two TGF-β1 expression groups (Supplementary Figure 1; pooling HR = 1.62, 95 % CI = 0.66–3.98, *p* = 0.29). These results suggest that high TGF-β1 expression could predict poor prognosis on DFS/RFS/MFS/PFS with COX univariate analysis by meta-analysis in HCC patients.

Publication bias was conducted via RevMan. Although the shape of funnel plots in OS and DFS/RFS/MFS/PFS outcomes did not meet global symmetry (Supplementary Figure 2), random-effect models were used, results were receivable and merit consideration. Furthermore, sensitivity tests were conducted during the meta-analysis, and elimination of any individual study did not alter the overall findings (Supplementary Table 3, Supplementary Table 4).

### Prognostic power of TGF-β1 expression in different sample types of HCC patients

#### Tissue

Subgroup analyses of HRs for OS and DFS/RFS/MFS/PFS among HCC patients in fixed- and random-effect models were carried out on different sample types: tissue, plasma/serum, and urine (Table 3, Table 4). Matching forest plots and funnel plots of sample type subgroups are in Supplementary Figures 3–11.

**Table 3:** Outcomes of subgroups analysis in fixed-effect models from different sample type of HCC patients

Subgroup	Univariate					Multivariable				
	N of S*	*I^2^* (%)	*p*’	HR (95% CI)	*p*-value	N of S	*I^2^* (%)	*p*’	HR (95% CI)	*p*-value
OS										
Tissue	7	36	0.16	1.79(1.49–2.15)	< 0.00001	4	76	0.006	1.86(1.49–2.32)	< 0.00001
Plasma/Serum	3	85	0.001	1.40(1.03–1.92)	0.03	2	83	0.02	1.66(1.07–2.58)	0.02
Urinary	2	0	0.67	1.29(0.94–1.75)	0.11	0	―	―	―	―
DFS/RFS/MFS/PFS										
Tissue	7	73	0.001	1.23(1.08–1.40)	0.002	2	88	0.003	1.95(1.12–3.41)	0.02
Plasma/Serum	1	―	―	1.75(1.13–2.71)	0.01	1	―	―	1.15(0.71–1.86)	0.56
Urinary	0	―	―	―	―	0	―	―	―	―

OS, overall survival; DFS, disease-free survival; RFS, relapse-free survival; MFS, metastasis-free survival; PFS, progression-free survival; N of S, number of studies; N of P, number of patients; HR, hazard ratio; CI, confidence interval;

―, there is no corresponding data presented;

*, Our analysis data from TCGA database, Test and Validation sets of Ruan's study were acted as three studies here.

**Table 4:** Outcomes of subgroups analysis in random-effect models from different sample type of HCC patients

Subgroup	Univariate					Multivariable				
	N of S	*I^2^* (%)	*p*’	HR (95% CI)	*p*-value	N of S	*I^2^* (%)	*p*’	HR (95% CI)	*p*-value
OS										
Tissue	7	36	0.16	1.90(1.47–2.46)	< 0.00001	4	76	0.006	2.44(1.40–4.27)	0.002
Plasma/Serum	3	85	0.001	1.73(0.73–4.07)	0.21	2	83	0.02	2.22(0.63–7.80)	0.21
Urinary	2	0	0.67	1.29(0.94–1.75)	0.11	0	―	―	―	―
DFS/RFS/MFS/PFS										
Tissue	7	73	0.001	1.59(1.15–2.19)	0.005	2	88	0.003	1.99(0.39–10.11)	0.41
Plasma/Serum	1	―	―	1.75(1.13–2.71)	0.01	1	―	―	1.15(0.71–1.86)	0.56
Urinary	0	―	―	―	―	0	―	―	―	―

OS, overall survival; DFS, disease-free survival; RFS, relapse-free survival; MFS, metastasis-free survival; PFS, progression-free survival; N of S, number of studies; N of P, number of patients; HR, hazard ratio; CI, confidence interval;

―, there is no corresponding data presented;

*, Our analysis data from TCGA database, Test and Validation sets of Ruan's study were acted as three studies here.

Data were derived from COX univariate analysis of seven studies. With a fixed-effect model, a shorter OS was observed in tissues with high TGF-β1 expression compared with those with low TGF-β1 expression (pooling HR = 1.79, 95% CI = 1.49–2.15, *p* < 0.00001; Table 3 and Supplementary Figure 3). Data were also derived from COX multivariate analysis of four studies. With a random-effect model, a shorter OS was observed in tissues of HCC patients with high TGF-β1 expression compared with those with low TGF-β1 expression (pooling HR = 2.44, 95% CI = 1.40–4.27, *p* = 0.002; Table 4, Supplementary Figure 6).

In addition, data were derived from COX multivariate analysis of two studies. With a random-effect model, there was no difference on DFS/RFS/MFS/PFS between the two TGF-β1 expression groups (pooling HR = 1.99, 95 % CI = 0.39–10.11, *p* = 0.41; Table 4, Supplementary Figure 10). Data were derived from COX univariate analysis of seven studies. With a random-effect model, a shorter DFS/RFS/MFS/PFS was observed in tissues of HCC patients with high TGF-β1 expression compared with those with low TGF-β1 expression (pooling HR = 1.59, 95% CI = 1.15–2.19, *p* = 0.005; Table 4 and Supplementary Figure 8).

#### Plasma/serum and urine

With a random-effect model, there was no difference on OS between high and low TGF-β1 expression in plasmas of HCC patients from both COX univariate analysis of three studies (pooling HR = 1.73, 95 % CI = 0.73–4.07, *p* = 0.21; Table 4 and Supplementary Figure 4) and COX multivariate analysis of two studies (pooling HR = 2.22, 95 % CI = 0.63–7.80, *p* = 0.21; Table 4 and Supplementary Figure 6). Similarly, with a fixed-effect model in a COX univariate analysis of 2 studies, there was no difference on OS between high and low TGF-β1 expression in urine of HCC patients (pooling HR = 1.29, 95% CI = 0.94–1.75, *p* = 0.11; Table 3 and Supplementary Figure 3).

## DISCUSSION

TGF-β1 expression can help primary carcinoma cells migrate and disseminate to distant sites [22–24]. This metastasis contributes to high mortality of hepatocellular carcinoma (HCC) [25–28], which is the third dominating cause of cancer-related deaths worldwide [29–32]. With so many people affected, it is important to study TGF-β1 in the development and progression of HCC. Some meta-analyses have reported that certain TGF-β1 polymorphisms (+869C/T and −509C/T) promote susceptibility to HCC [33–36], but clinical significance and prognostic value of TGF-β1 expression in HCC has remained uncharacterized.

We evaluated the prognostic value of TGF-β1 expression in 12 articles and our TCGA analyzing data with 2,021 total HCC patients via implementing a meta-analysis. We found that high TGF-β1 expression can act as an independent indicator of unfavorable prognosis on OS from original univariate and multivariate analysis. High TGF-β1 expression can predict a worse DFS/RFS/PFS in meta-analysis from original univariate analysis, while multivariate analysis yielded no difference on DFS/PFS between the groups of high and low TGF-β1 expression.

We first analyzed a dataset of 423 HCC patients from TCGA, and found that high TGF-β1 expression could indicate an unfavorable prognosis on OS, but not on RFS. The results were consistent with our meta-analysis on OS. The different clinical effects of high TGF-β1 expression in the same tumor type from different studies may be due to variant cut-off values of TGF-β1 expression between different studies. The complex environment of cancer cells and the duality of TGF-β1 in cancers are other possible causes. TGF-β1 can act as an epithelium (including hepatocellular) cellular proliferative depressor, a tumor accelerator, or both, depending on the cell environment [37, 38].

Although there was a certain but acceptable heterogeneity in OS and DFS/RFS/PFS studies, the outcomes of meta-analysis still deserve to be considered. Several reasons could have contributed to the heterogeneity, including the constituent ratio of patients’ age, detection method, and a diverse source of samples in each study. For example, samples in two articles were from urine [16, 20], three from plasma/serum [10, 13, 17], and seven from tumor tissue [11, 12, 14, 15, 18, 19, 21]. Secondly, there were variable definitions of the cut-off value among these publications. No relevant studies to investigate the uniform criterion of TGF-β1 positive or high expression, which could be an important source of potential bias. Thirdly, all the incorporated publications were completely implemented in Asia. Lastly, the original individual information of HCC patients was not available for all studies, which also likely led to the heterogeneity of our analysis.

Some limitations existed in our study. All the studies were from Asia, which possibly resulted in regional bias. The articles incorporated into our analyses were all published in English, so the language bias appeared. Selective reporting was observed in some publications, which led to the loss of some meritorious data. For example, the pooled HRs for DFS/RFS/MFS/PFS were displayed in fewer studies compared to HRs for OS. The HRs that were evaluated indirectly might be less reliable than those acquired directly from published data. Finally, if these studies could have provided patients’ individual information, our meta-analysis would have been much more precise.

In summary, both data mining results from the TCGA database and meta-analysis results from published studies definitively presented a negative prognostic effect of high TGF-β1 expression on the OS in HCC patients. Our results may provide an insight for the prognostic prediction of HCC patients. Further preclinical and clinical research with larger samples and open individual data of patients are required to validate the prognostic significance of TGF-β1 expression in HCC patients.

## MATERIALS AND METHODS

### Data extraction from TCGA database

An independent dataset including information on mRNA expression and clinical features of HCC patients (*n* = 423 for TCGA liver hepatocellular carcinoma, gene expression by RNAseq with IlluminaHiSeq) was obtained from the UCSC Cancer Genomics Browser of TCGA (https://genome-cancer.soe.ucsc.edu). We analyzed the differences of clinicopathologic variables between two groups: high TGF-β1 expression and low TGF-β1 expression. We also evaluated the prognostic influence of TGF-β1 expression on OS and RFS in this HCC cohort.

### Kaplan-meier survival analysis and COX regression analysis

Differences between the clinicopathological data of higher TGF-β1 expression and lower TGF-β1 expression was assessed by the Chi-squared test. For survival analysis, OS was calculated from the day of diagnosis to the day of death or last follow-up, while RFS was defined as the time from the day of the first complete remission to the day of first relapse or death [39–42]. Survival curves were established using the Kaplan-Meier approach, with log-rank tests applied to appraise the differences between the groups. Hazard ratios (HRs) were produced using COX's proportional hazards model. COX Univariate and multivariate models for the prognostic effect of TGF-β1 expression on OS/RFS in HCC patients from the TCGA were analyzed. SPSS 17.0 software (IBM, Chicago, USA) was applied to conduct statistical analysis, and a two-sided *p-value* < 0.05 was regarded as statistical significance. All survival-related figures were plotted in GraphPad Prism 5 (GraphPad, La Jolla, USA).

### Literature search for meta-analysis

Literatures search was retrieved from PubMed, Web of Knowledge, Embase, ClinicalTrials, and the Cochrane Library with the following search terms (mainly from MeSH Term and its corresponding Entry Terms): TGF-beta1, Transforming Growth Factor beta 1, TGF-beta-1, TGF beta 1, TGF-β1, Transforming Growth Factor β1, TGF-β-1, TGF β1, Transforming Growth Factor beta 1 Latency Associated Peptide, TGF-beta1 Latency-Associated Protein, Latency-Associated Protein, TGF-beta1, TGF beta1 Latency Associated Protein, TGF-beta1LAP, TGF beta1LAP; AND Liver Neoplasms, Neoplasms, Liver, Liver Neoplasm, Neoplasm, Liver, Hepatic Neoplasms, Neoplasms, Hepatic, Hepatic Neoplasm, Neoplasm, Hepatic, Cancer of Liver, Cancer, Hepatocellular, Hepatocellular Cancer, HCC, Cancers, Hepatocellular, Hepatocellular Cancers, Cancer, Hepatic, Hepatic Cancer, Cancers, Hepatic, Hepatic Cancers, Cancer, Liver, Liver Cancer, Cancers, Liver, Liver Cancers, Cancer of the Liver; AND prognostic, prognosis, prognoses, outcome, outcomes, mortality, survival, overall survival, OS, disease-free survival, DFS, relapse-free survival, recurrence-free survival, RFS, metastasis-free survival, MFS, progression-free survival, or PFS.

### Study selection, data extraction, and quality assessment of meta-analysis

No related review protocol has existed or been registered. Inclusion criteria: (1) studies were full papers in English prior to February 10, 2017; (2) Original articles as cohort studies; (3) Patients were grouped in terms of the expression levels of TGF-β1; (4) Focused on prognostic effect of TGF-β1 expression on patients with liver cancer; (5) Offered data on survival including OS and/or DFS/RFS/MFS/PFS. Exclusion criteria: (1) Duplicate publications; (2) Conference abstracts, meta-analysis, reviews, letters, comments, expert opinions and case reports; (3) Being not yet published in English; (4) Non-human experiments; (5) Studies without qualified data. Repetitive literature was managed and removed by Endnote X4.

Two researchers independently inspected all literature that satisfied the inclusion criteria, and the divergences between reviewers were settled through symposium. Information including first author, publication year, region, median age, sample size, gender distribution, cancer type, tumor stage, detection method, and follow-up time were extracted from each eligible study. Matching HRs with 95% CI for OS and DFS/RFS/MFS/PFS were calculated using COX univariable and multivariable models containing original data, or data extracted from Kaplan-Meier curves, as previously described [43, 44].

The methodological quality of included literature was appraised via the Newcastle-Ottawa Scale (NOS) [45]. The NOS is composed of three dimensions: selection, comparability, and exposure or outcome. Up to 4, 2, and 3 stars are given for the three dimensions respectively, with a total maximum score of 9 stars. Using the NOS, the quality of these studies was classified into three tiers: high quality (7–9 stars), intermediate quality (4–6 stars), and low quality (1–3 stars) [45–47].

### Statistics of meta-analysis

Meta-analysis was conducted with Review Manager (RevMan) software (version 5.3.5; the Nordic Cochrane Centre, Copenhagen, Denmark). The prognostic role of TGF-β1 expression on OS and/or DFS/RFS/MFS/PFS was assessed by the pooled HRs and their matching 95% CI with the inverse variance method. Statistical heterogeneity was assessed by the chi-squared test (the significance of heterogeneity was artificially expressed as *p’*-value to distinguish from the significance of outcomes) and *I*^2^ statistics. When there was no significant heterogeneity (*p’*-value > 0.1 and *I*^2^ < 50%), the pooled HRs were assessed by fixed-effect models. Otherwise, random-effect models were utilized to enhance the stability of the meta-analysis by providing a conservative standard error and wider confidence interval. Publication bias was appraised by Begg funnel plot and Egger's test.

## SUPPLEMENTARY MATERIALS FIGURES AND TABLES





## References

[1] Ferlay J, Soerjomataram I, Dikshit R, Eser S, Mathers C, Rebelo M, Parkin DM, Forman D, Bray F (2015). Cancer incidence and mortality worldwide: sources, methods and major patterns in GLOBOCAN 2012. Int J Cancer.

[2] Liu ZM, Tseng HY, Tsai HW, Su FC, Huang HS (2015). Transforming growth factor beta-interacting factor-induced malignant progression of hepatocellular carcinoma cells depends on superoxide production from Nox4. Free Radic Biol Med.

[3] Gravitz L (2014). Liver cancer. Nature.

[4] Allemani C, Weir HK, Carreira H, Harewood R, Spika D, Wang XS, Bannon F, Ahn JV, Johnson CJ, Bonaventure A, Marcos-Gragera R, Stiller C, Azevedo e Silva G (2015). Global surveillance of cancer survival 1995-2009: analysis of individual data for 25,676,887 patients from 279 population-based registries in 67 countries (CONCORD-2). Lancet.

[5] Roberts AB, Anzano MA, Lamb LC, Smith JM, Sporn MB (1981). New class of transforming growth factors potentiated by epidermal growth factor: isolation from non-neoplastic tissues. Proc Natl Acad Sci U S A.

[6] Giannelli G, Villa E, Lahn M (2014). Transforming growth factor-beta as a therapeutic target in hepatocellular carcinoma. Cancer Res.

[7] Mazzocca A, Fransvea E, Dituri F, Lupo L, Antonaci S, Giannelli G (2010). Down-Regulation of Connective Tissue Growth Factor by Inhibition of Transforming Growth Factor beta Blocks the Tumor-Stroma Cross-Talk and Tumor Progression in Hepatocellular Carcinoma. Hepatology.

[8] Peng L, Zhou Y, Dong L, Chen RQ, Sun GY, Liu T, Ran WZ, Fang X, Jiang JX, Guan CX (2016). TGF-beta1 Upregulates the Expression of Triggering Receptor Expressed on Myeloid Cells 1 in Murine Lungs. Sci Rep.

[9] Li S, Yang F, Ren X (2015). Immunotherapy for hepatocellular carcinoma. Drug Discov Ther.

[10] Lin TH, Shao YY, Chan SY, Huang CY, Hsu CH, Cheng AL (2015). High Serum Transforming Growth Factor-beta1 Levels Predict Outcome in Hepatocellular Carcinoma Patients Treated with Sorafenib. Clin Cancer Res.

[11] Wang Y, Liu T, Tang W, Deng B, Chen Y, Zhu J, Shen X (2016). Hepatocellular Carcinoma Cells Induce Regulatory T Cells and Lead to Poor Prognosis via Production of Transforming Growth Factor-beta1. Cell Physiol Biochem.

[12] Ji F, Fu SJ, Shen SL, Zhang LJ, Cao QH, Li SQ, Peng BG, Liang LJ, Hua YP (2015). The prognostic value of combined TGF-beta1 and ELF in hepatocellular carcinoma. BMC Cancer.

[13] Chao Y, Wu CY, Kuo CY, Wang JP, Luo JC, Kao CH, Lee RC, Lee WP, Li CP (2013). Cytokines are associated with postembolization fever and survival in hepatocellular carcinoma patients receiving transcatheter arterial chemoembolization. Hepatol Int.

[14] Turato C, Vitale A, Fasolato S, Ruvoletto M, Terrin L, Quarta S, Ramirez Morales R, Biasiolo A, Zanus G, Zali N, Tan PS, Hoshida Y, Gatta A (2014). SERPINB3 is associated with TGF-beta1 and cytoplasmic beta-catenin expression in hepatocellular carcinomas with poor prognosis. Br J Cancer.

[15] Xiang ZL, Zeng ZC, Tang ZY, Fan J, He J, Zeng HY, Zhu XD (2011). Potential prognostic biomarkers for bone metastasis from hepatocellular carcinoma. Oncologist.

[16] Tsai JF, Chuang LY, Jeng JE, Yang ML, Chang WY, Hsieh MY, Lin ZY, Tsai JH (1997). Clinical relevance of transforming growth factor-beta 1 in the urine of patients with hepatocellular carcinoma. Medicine (Baltimore).

[17] Okumoto K, Hattori E, Tamura K, Kiso S, Watanabe H, Saito K, Saito T, Togashi H, Kawata S (2004). Possible contribution of circulating transforming growth factor-beta1 to immunity and prognosis in unresectable hepatocellular carcinoma. Liver Int.

[18] Gai X, Tu K, Lu Z, Zheng X (2014). MRC2 expression correlates with TGFbeta1 and survival in hepatocellular carcinoma. Int J Mol Sci.

[19] Chen Z, Xie B, Zhu Q, Xia Q, Jiang S, Cao R, Shi L, Qi D, Li X, Cai L (2013). FGFR4 and TGF-beta1 expression in hepatocellular carcinoma: correlation with clinicopathological features and prognosis. Int J Med Sci.

[20] Tsai JF, Jeng JE, Chuang LY, Yang ML, Ho MS, Chang WY, Hsieh MY, Lin ZY, Tsai JH (1997). Elevated urinary transforming growth factor-beta1 level as a tumour marker and predictor of poor survival in cirrhotic hepatocellular carcinoma. Br J Cancer.

[21] Ruan H, Wang T, Yang C, Jin G, Gu D, Deng X, Wang C, Qin W, Jin H (2016). Co-expression of LASS2 and TGF-beta1 predicts poor prognosis in hepatocellular carcinoma. Sci Rep.

[22] Gupta S, Maitra A. EMT (2016). Matter of Life or Death?. Cell.

[23] David CJ, Huang YH, Chen M, Su J, Zou Y, Bardeesy N, Iacobuzio-Donahue CA, Massague J (2016). TGF-beta Tumor Suppression through a Lethal EMT. Cell.

[24] Nieto MA, Huang RY, Jackson RA, Thiery JP (2016). EMT: 2016. Cell.

[25] Zhao J, Wu J, Cai H, Wang D, Yu L, Zhang WH (2016). E3 Ubiquitin Ligase Siah-1 is Down-regulated and Fails to Target Natural HBx Truncates for Degradation in Hepatocellular Carcinoma. J Cancer.

[26] Huan H, Wen X, Chen X, Wu L, Liu W, Habib NA, Bie P, Xia F (2016). C/EBPalpha Short-Activating RNA Suppresses Metastasis of Hepatocellular Carcinoma through Inhibiting EGFR/beta-Catenin Signaling Mediated EMT. PLoS One.

[27] Wong CM, Wong CC, Lee JM, Fan DN, Au SL, Ng IO (2012). Sequential alterations of microRNA expression in hepatocellular carcinoma development and venous metastasis. Hepatology.

[28] Fang JH, Zhou HC, Zhang C, Shang LR, Zhang L, Xu J, Zheng L, Yuan Y, Guo RP, Jia WH, Yun JP, Chen MS, Zhang Y (2015). A novel vascular pattern promotes metastasis of hepatocellular carcinoma in an epithelial-mesenchymal transition-independent manner. Hepatology.

[29] Njei B, Rotman Y, Ditah I, Lim JK (2015). Emerging trends in hepatocellular carcinoma incidence and mortality. Hepatology.

[30] Fan QM, Jing YY, Yu GF, Kou XR, Ye F, Gao L, Li R, Zhao QD, Yang Y, Lu ZH, Wei LX (2014). Tumor-associated macrophages promote cancer stem cell-like properties via transforming growth factor-beta1-induced epithelial-mesenchymal transition in hepatocellular carcinoma. Cancer Lett.

[31] Zhou X, Zhang CZ, Lu SX, Chen GG, Li LZ, Liu LL, Yi C, Fu J, Hu W, Wen JM, Yun JP (2015). miR-625 suppresses tumour migration and invasion by targeting IGF2BP1 in hepatocellular carcinoma. Oncogene.

[32] Jiang G, Zhang L, Zhu Q, Bai D, Zhang C, Wang X (2016). CD146 promotes metastasis and predicts poor prognosis of hepatocellular carcinoma. J Exp Clin Cancer Res.

[33] Lu WQ, Qiu JL, Huang ZL, Liu HY (2016). Enhanced circulating transforming growth factor beta 1 is causally associated with an increased risk of hepatocellular carcinoma: a mendelian randomization meta-analysis. Oncotarget.

[34] Toshikuni N, Matsue Y, Minato T, Hayashi N, Tsutsumi M (2016). Association between transforming growth factor-beta1 -509 C>T variants and hepatocellular carcinoma susceptibility: a meta-analysis. Neoplasma.

[35] Guo Y, Zang C, Li Y, Yuan L, Liu Q, Zhang L, Li S (2013). Association between TGF-beta1 polymorphisms and hepatocellular carcinoma risk: a meta-analysis. Genet Test Mol Biomarkers.

[36] Zhang CF, Wang ZW, Hou MX, Li K, Zhou X, Xia YH (2012). Transforming growth factor beta1-509C/T and +869T/C polymorphisms on the risk of upper digestive tract cancer: a meta-analysis based on 10,917 participants. Ann Hum Genet.

[37] Deane NG, Lee H, Hamaamen J, Ruley A, Washington MK, LaFleur B, Thorgeirsson SS, Price R, Beauchamp RD (2004). Enhanced tumor formation in cyclin D1 x transforming growth factor beta1 double transgenic mice with characterization by magnetic resonance imaging. Cancer Res.

[38] Derynck R, Akhurst RJ, Balmain A (2001). TGF-beta signaling in tumor suppression and cancer progression. Nat Genet.

[39] Wang F, Zhang SD, Xu HM, Zhu JH, Hua RX, Xue WQ, Li XZ, Wang TM, He J, Jia WH (2016). XPG rs2296147 T>C polymorphism predicted clinical outcome in colorectal cancer. Oncotarget.

[40] Yuan XQ, Zhang DY, Yan H, Yang YL, Zhu KW, Chen YH, Li X, Yin JY, Li XL, Zeng H, Chen XP (2016). Evaluation of DNMT3A genetic polymorphisms as outcome predictors in AML patients. Oncotarget.

[41] Yuan XQ, Peng L, Zeng WJ, Jiang BY, Li GC, Chen XP (2016). DNMT3A R882 Mutations Predict a Poor Prognosis in AML: A Meta-Analysis From 4474 Patients. Medicine (Baltimore).

[42] Peng L, Yuan XQ, Liu ZY, Li WL, Zhang CY, Zhang YQ, Pan X, Chen J, Li YH, Li GC (2017). High lncRNA H19 expression as prognostic indicator: data mining in female cancers and polling analysis in non-female cancers. Oncotarget.

[43] Tierney JF, Stewart LA, Ghersi D, Burdett S, Sydes MR (2007). Practical methods for incorporating summary time-to-event data into meta-analysis. Trials.

[44] Parmar MK, Torri V, Stewart L (1998). Extracting summary statistics to perform meta-analyses of the published literature for survival endpoints. Stat Med.

[45] Wells GA, Shea B, O'Connell D, Peterson J, Welch V, Losos M, Tugwell P ((2012)). The Newcastle-Ottawa Scale (NOS) for assessing the quality of nonrandomised studies in meta-analyses.

[46] Tie R, Zhang T, Fu H, Wang L, Wang Y, He Y, Wang B, Zhu N, Fu S, Lai X, Shi J, Huang H (2014). Association between DNMT3A mutations and prognosis of adults with de novo acute myeloid leukemia: a systematic review and meta-analysis. PLoS One.

[47] Xie S, Xu H, Shan X, Liu B, Wang K, Cai Z (2015). Clinicopathological and prognostic significance of survivin expression in patients with oral squamous cell carcinoma: evidence from a meta-analysis. PLoS One.

