# Supplementary material for: High TGF-β1 expression predicts poor disease prognosis in hepatocellular carcinoma patients
Source: Oncotarget. 2017 Mar 13;8(21):34387–97. doi: 10.18632/oncotarget.16166 (PMC5470976; doi:10.18632/oncotarget.16166)
Supplement: Supplementary file 1 [file oncotarget-08-34387-s001.pdf]

High TGF-β1 expression predicts poor disease prognosis in hepatocellular carcinoma patients

Supplementary Materials

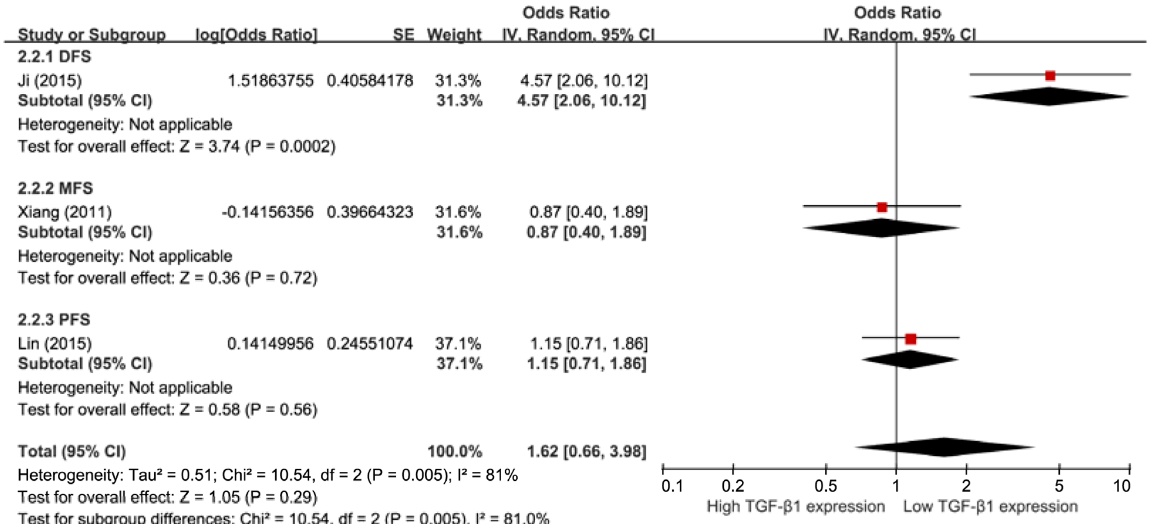

Supplementary Figure 1: Meta-analysis of the HRs with 95% CI for DFS from multivariate analysis in HCC. The size of the blocks or diamonds represents the weight for the random-effect model in the meta-analysis. HR > 1 indicates that high TGF-β1 expression is correlated with a more unfavorable disease-free survival (DFS).

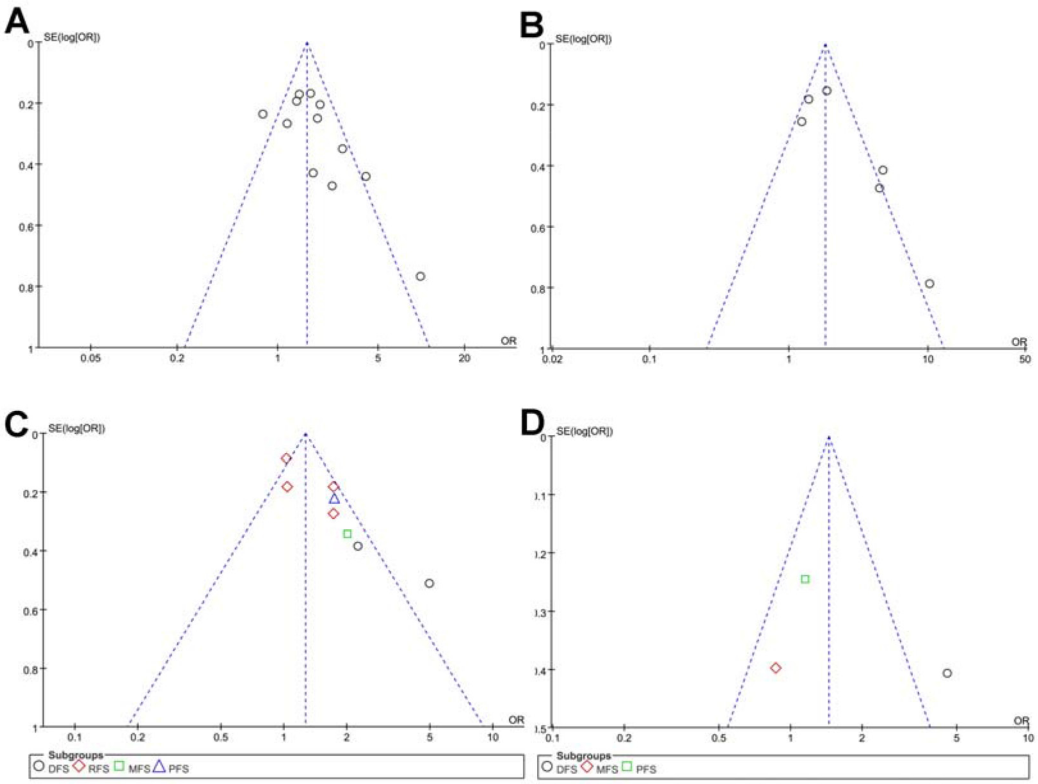

Supplementary Figure 2: Funnel plots on HRs of TGF-β1 expression in HCC patients. Funnel plots illustrated a limited symmetry on HRs for overall survival (OS) from univariate analysis (**A**) and multivariate analysis (**B**) in HCC patients. Funnel plots illustrated a limited symmetry on HRs for disease-free survival (DFS)/relapse-free survival (RFS)/metastasis-free survival (MFS)/ progression-free survival (PFS) from COX univariate analysis (**C**) and COX multivariate analysis (**D**) in HCC patients.

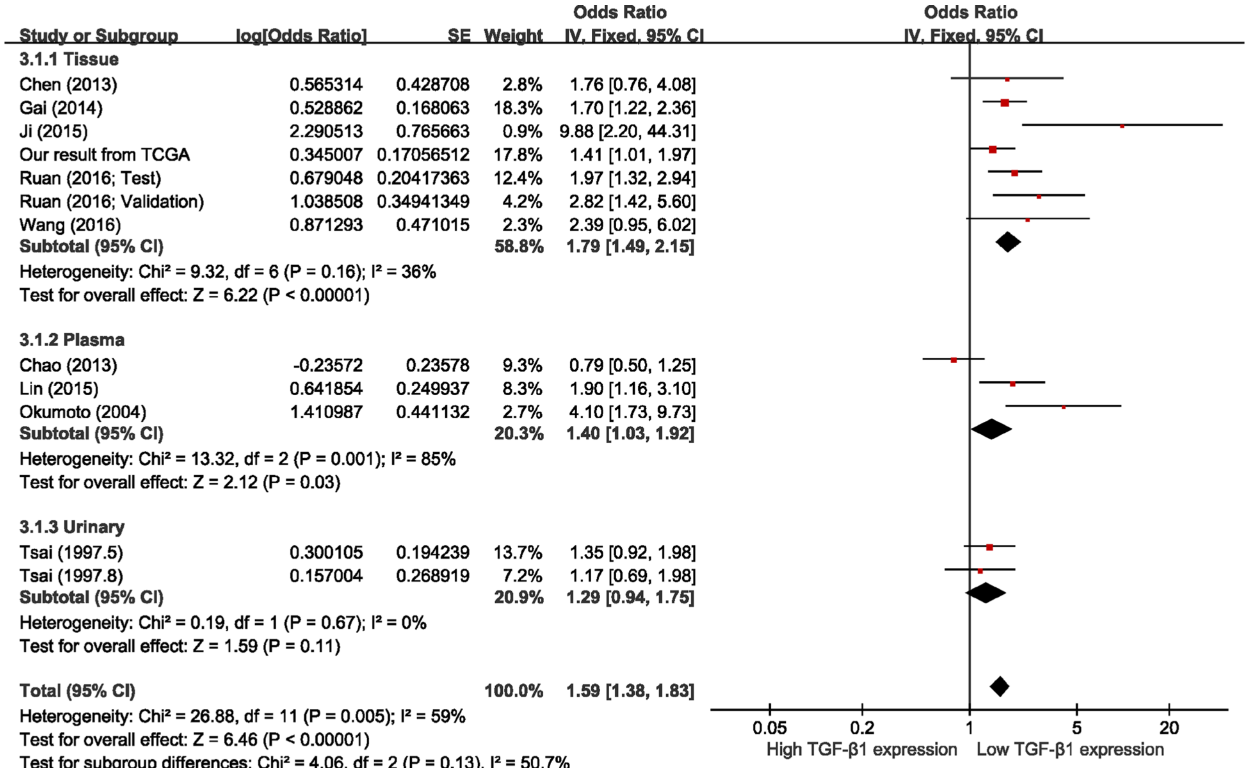

Supplementary Figure 3: Forest plots on OS from univariate analysis in HCC patients of different sample types. The size of the blocks or diamonds represents the weight for the fixed-effect model in the meta-analysis. HR > 1 indicates that high TGF-β1 expression is associated with a shorter overall survival (OS).

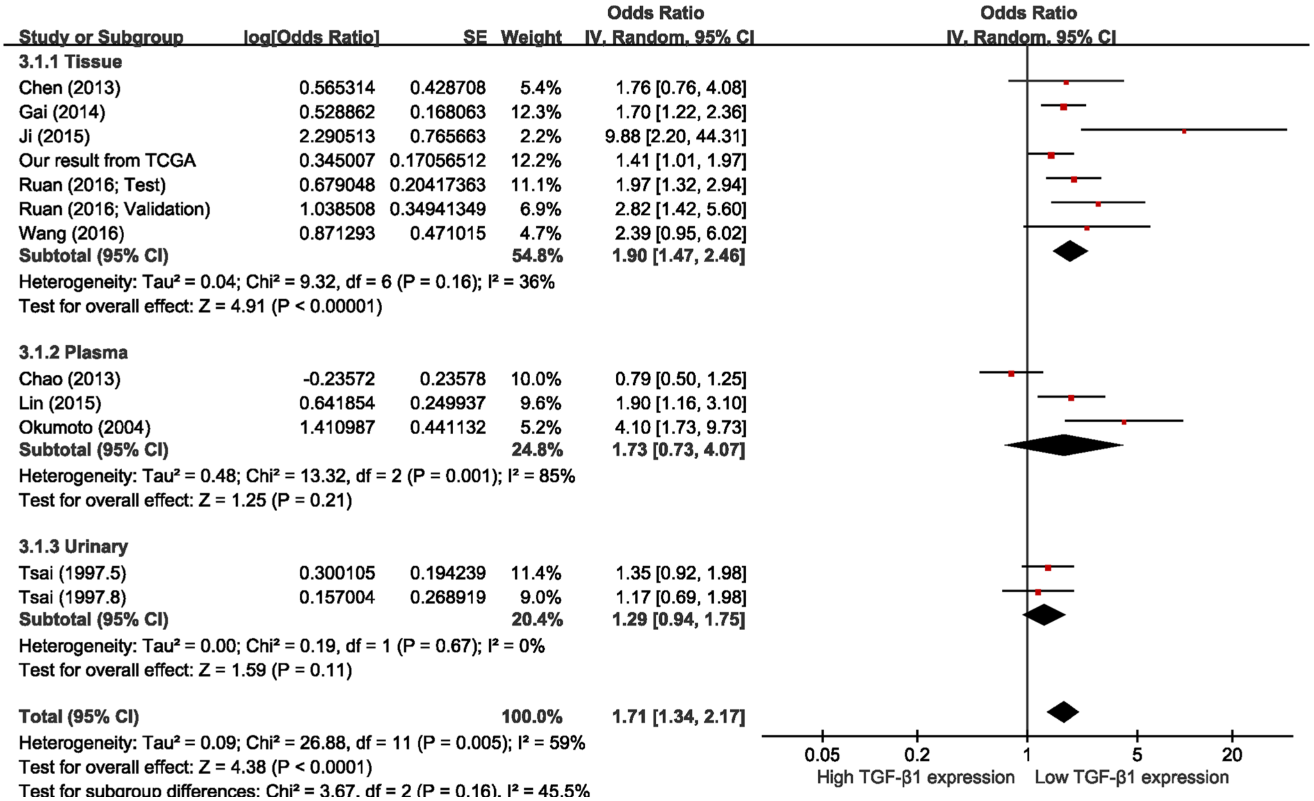

Supplementary Figure 4: Forest plots on OS from univariate analysis in HCC patients of different sample types. The size of the blocks or diamonds represents the weight for the random-effect model in the meta-analysis. HR>1 indicates that high TGF-β1 expression is associated with a shorter overall survival (OS).

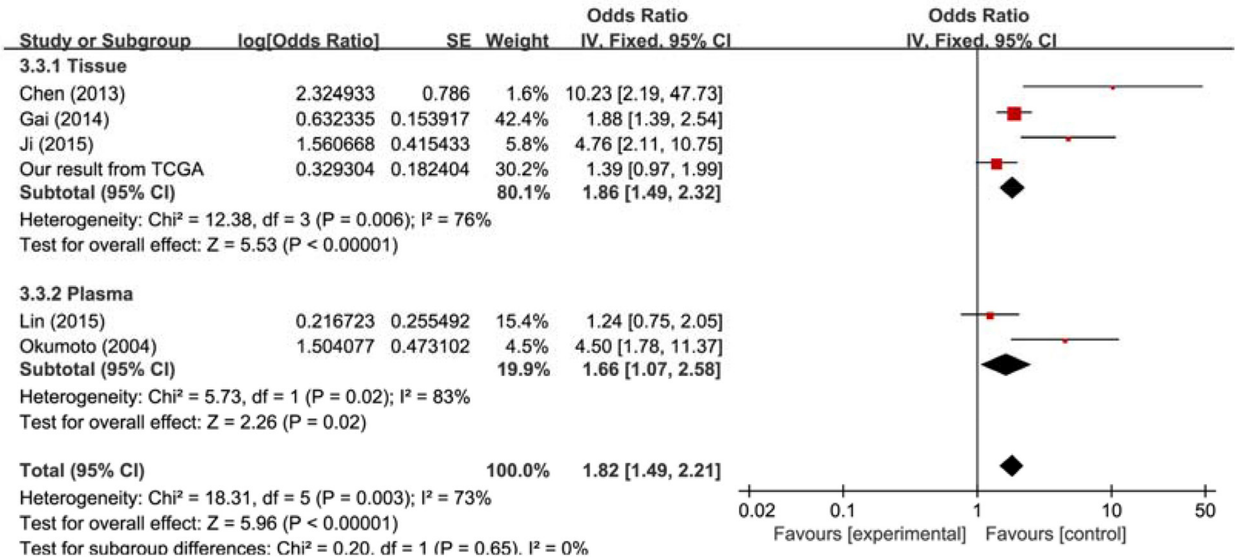

Supplementary Figure 5: Forest plots on OS from multivariate analysis in HCC patients of different sample types. The size of the blocks or diamonds represents the weight for the fixed-effect model in the meta-analysis. HR>1 indicates that high TGF-β1 expression is associated with a shorter overall survival (OS).

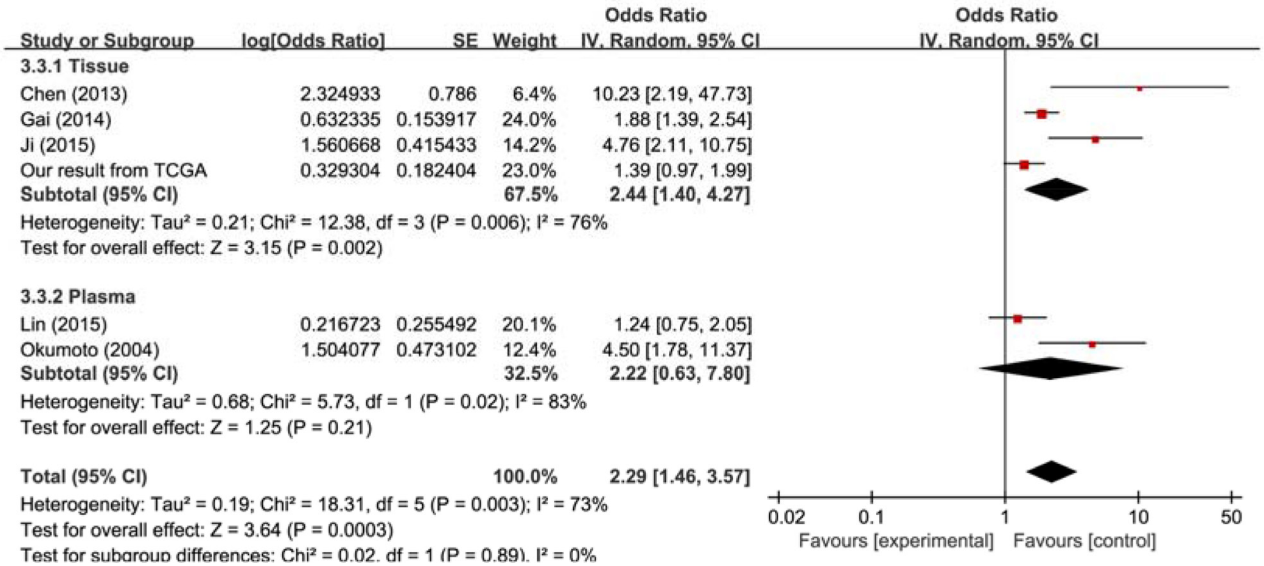

Supplementary Figure 6: Forest plots on OS from multivariate analysis in HCC patients of different sample types. The size of the blocks or diamonds represents the weight for the random-effect model in the meta-analysis. HR>1 indicates that high TGF-β1 expression is associated with a shorter overall survival (OS).

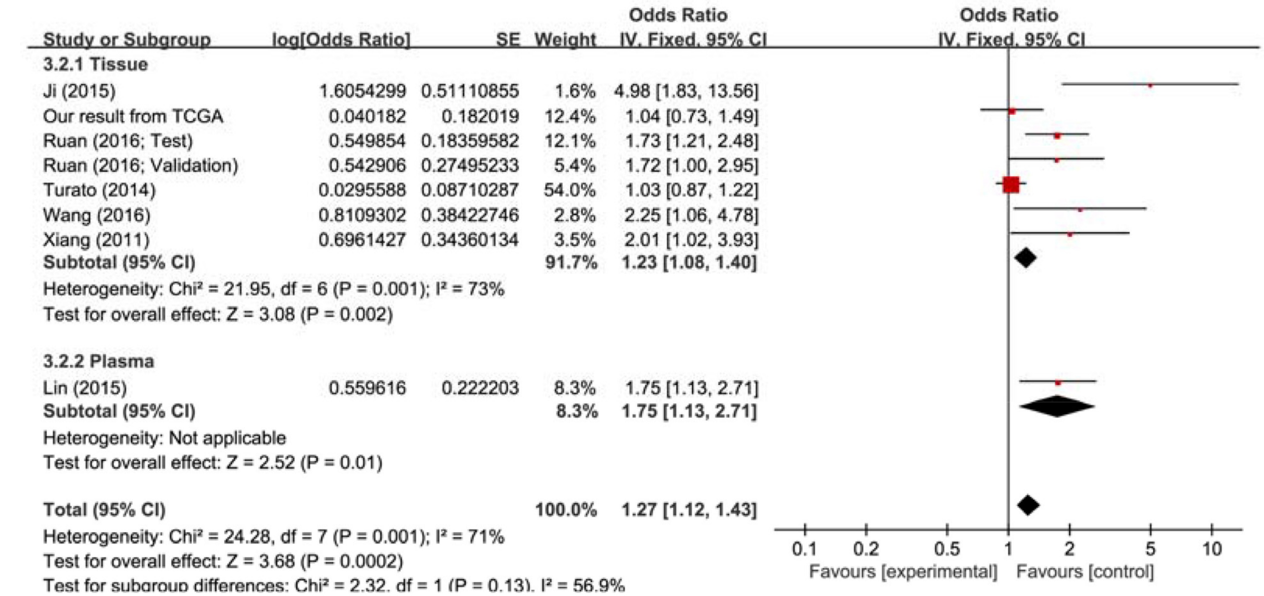

Supplementary Figure 7: Forest plots on DFS/RFS/MFS/PFS from univariate analysis in HCC patients of different sample types. The size of the blocks or diamonds represents the weight for the fixed-effect model in the meta-analysis. HR>1 indicates that high TGF-β1 expression is associated with a shorter disease-free survival (DFS)/relapse-free survival (RFS)/metastasis-free survival (MFS)/progression-free survival (PFS).

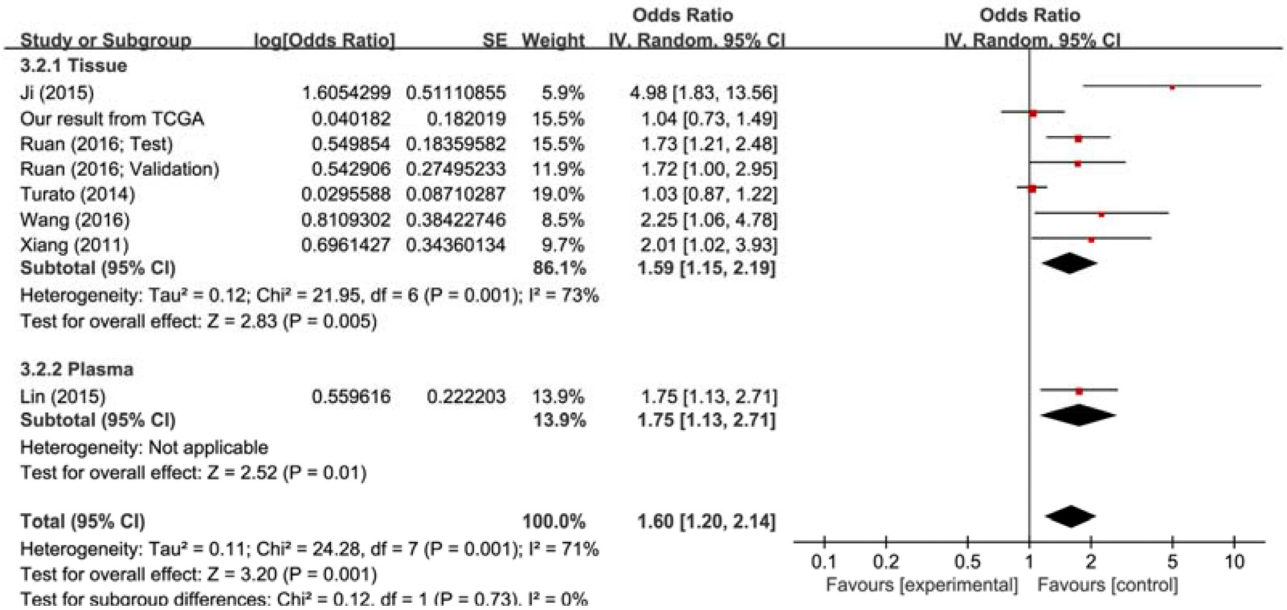

Supplementary Figure 8: Forest plots on DFS/RFS/MFS/PFS from univariate analysis in HCC patients of different sample types. The size of the blocks or diamonds represents the weight for the random-effect model in the meta-analysis. HR > 1 indicates that high TGF-β1 expression is associated with a shorter disease-free survival (DFS)/relapse-free survival (RFS)/metastasis-free survival (MFS)/progression-free survival (PFS).

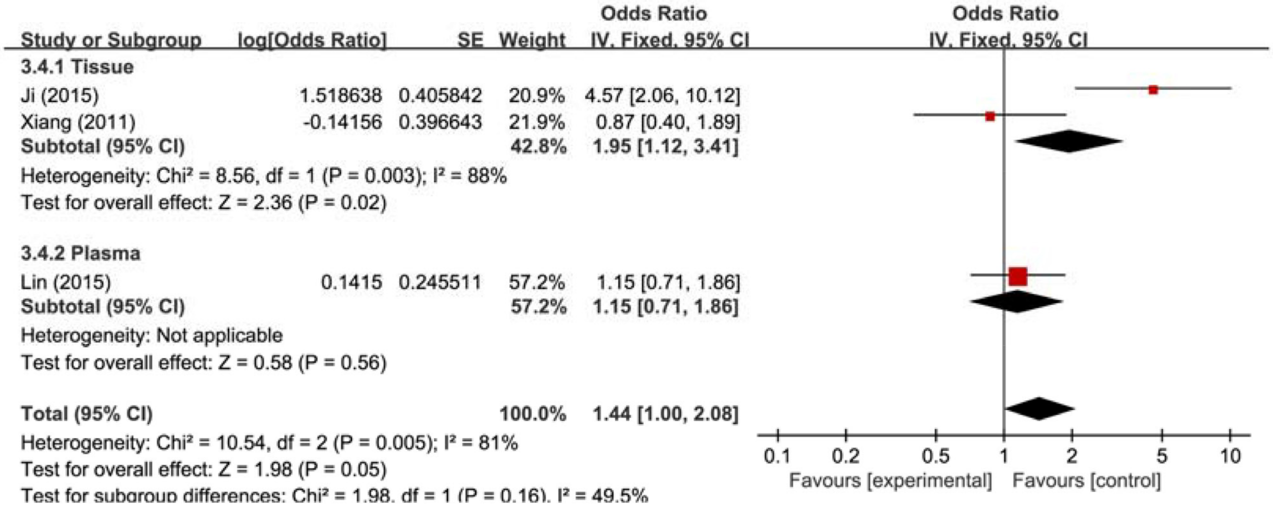

Supplementary Figure 9: Forest plots on DFS/MFS/PFS from multivariate analysis in HCC patients of different sample types. The size of the blocks or diamonds represents the weight for the fixed-effect model in the meta-analysis. HR>1 indicates that high TGF-β1 expression is associated with a shorter disease-free survival (DFS)/metastasis-free survival (MFS)/progression-free survival (PFS).

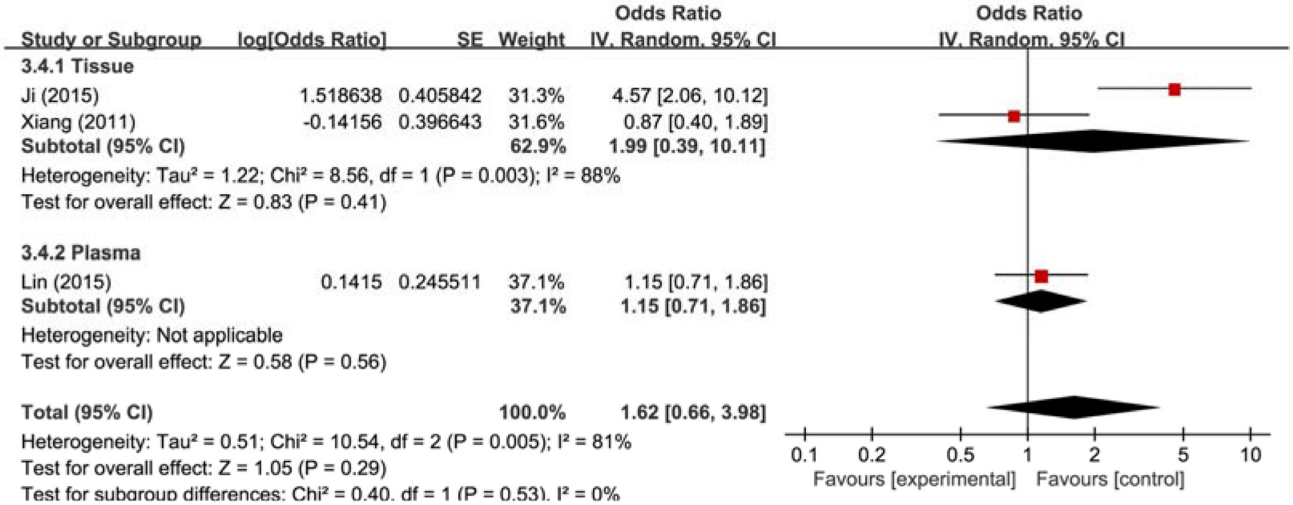

Supplementary Figure 10: Forest plots on DFS/MFS/PFS from multivariate analysis in HCC patients of different sample types. The size of the blocks or diamonds represents the weight for the random-effect model in the meta-analysis. HR>1 indicates that high TGF-β1 expression is associated with a shorter disease-free survival (DFS)/metastasis-free survival (MFS)/progression-free survival (PFS).

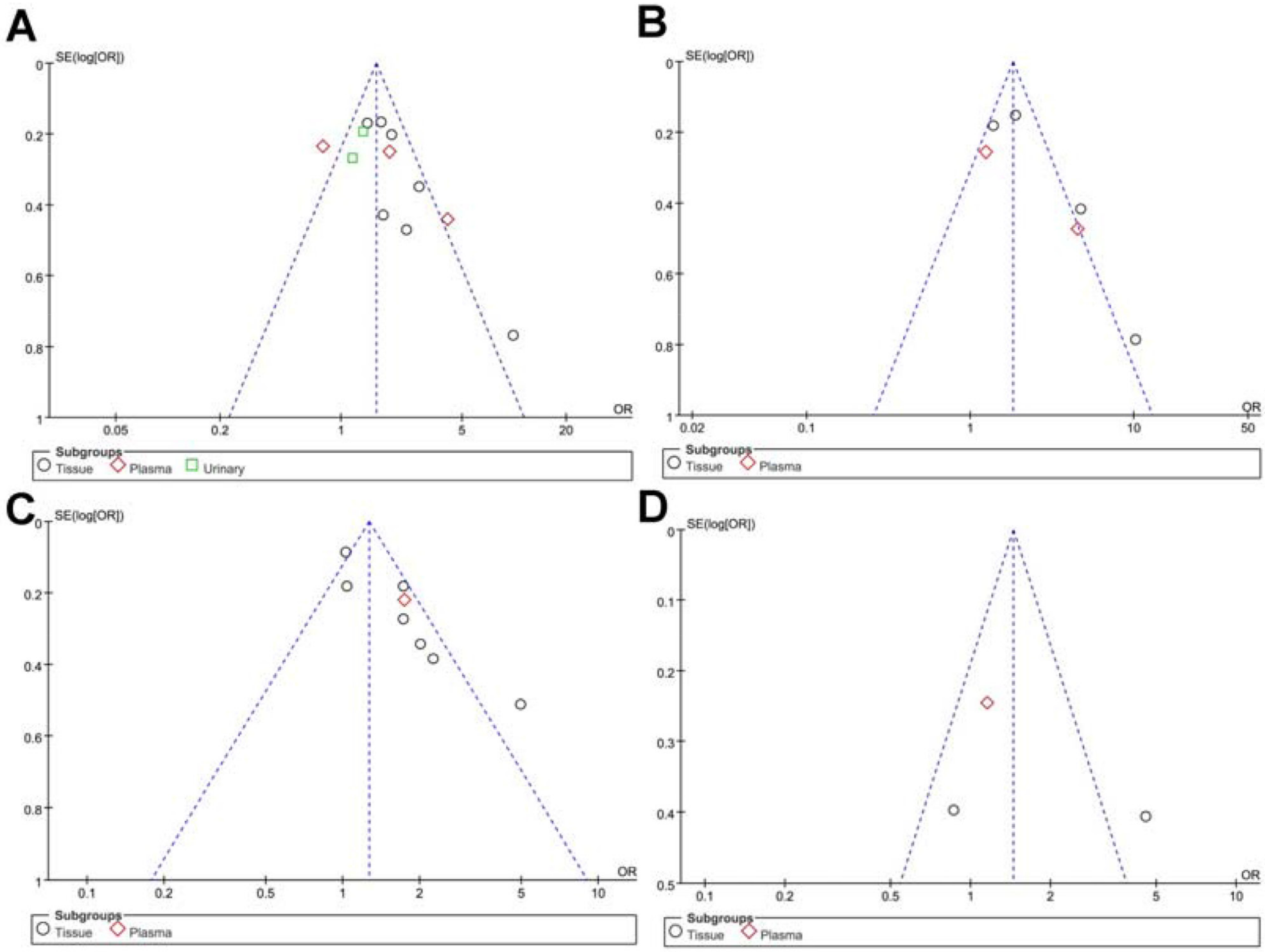

Supplementary Figure 11: Funnel plots on HRs of TGF-β1 expression in HCC patients of different sample types. Funnel plots illustrated a limited symmetry on HRs for overall survival (OS) from univariate analysis (**A**) and multivariate analysis (**B**) in HCC patients. Funnel plots illustrated a limited symmetry on HRs for disease-free survival (DFS)/relapse-free survival (RFS)/metastasis-free survival (MFS)/progression-free survival (PFS) from COX univariate analysis (**C**) and COX multivariate analysis (**D**) in HCC patients.

Supplementary Table 1: The clinic-pathological characteristics of 423 HCC patients according to TGF-β1 mRNA expression from. See Supplementary_Table_1

Supplementary Table 2: Univariate and multivariate analysis of clinic pathologic factors for relapse-free survival of 423 HCC patients from TCGA

Risk factors	Univariate analysis			Multivariate analysis		
	HR	95 % CI	*p*	HR	95 % CI	*p*
TGF-β1 expression (high vs. low)	1.041	0.729–1.488	0.825			
Gender (male vs. female)	0.853	0.592–1.229	0.392			
Age (> 62 vs. ≦ 62)	1.229	0.858–1.762	0.260			
Weight (> 35 vs. ≦ 35 kg)	1.300	0.883–1.915	0.184			
Height (> 1.68 vs. ≦ 168 cm)	1.263	0.857–1.861	0.238			
BMI (≥ 25 vs. < 25)	0.048	0.000–75.357	0.419			
BMI' (> vs. ≦ 12.22363946)	1.070	0.723–1.582	0.736			
Clinical stage (III–IV vs. I–II)	2.332	1.547–3.516	< 0.0001	2.769	1.752–4.376	< 0.0001
Grade (G3–4 vs. G1–2)	1.177	0.813–1.705	0.387			
platelet (> 212 vs. ≦ 212)	1.459	1.001–2.127	0.049	1.119	0.719–1.741	0.622
Albumin (> 4 vs. ≦ 4)	0.809	0.557–1.175	0.266			
Alpha fetoprotein (> 16 vs. ≦ 16)	1.264	0.840–1.902	0.261			
Serum creatinine (> 0.9 vs. ≦ 0.9)	0.798	0.542–1.173	0.250			
Prothrombin time (> 1.1 vs. ≦ 1.1)	1.337	0.918–1.948	0.130			
Total bilirubn (> 0.7 vs. ≦ 0.7)	0.898	0.605–1.334	0.594			
Liver fibrosis ishak score category (fibrosis vs. no fibrosis)	0.656	0.159–2.712	0.561			
Vascular tumor cell invasion (positive vs. negative)	1.481	0.998–2.196	0.051	1.017	0.642–1.611	0.990

Supplementary Table 3: Sensitivity tests for survival from COX univariate analysis in HCC patients

Exclusion study	OS			DFS/RFS/MFS/PFS		
	I^2 (%)	*p*'	HR(95%CI)	I2 (%)	*p*'	HR(95%CI)
No exclusion	59	0.005	1.71(1.34–2.17)	71	0.001	1.60(1.20–2.14)
Chao 2012	42	0.07	1.81(1.46–2.23)	—	—	—
Chen 2013	63	0.003	1.71(1.33–2.21)	—	—	—
Gai 2014	63	0.003	1.74(1.32–2.29)	—	—	—
Ji 2015	53	0.02	1.63(1.31–2.03)	65	0.009	1.46(1.13–1.90)
Lin 2015	62	0.003	1.70(1.31–2.21)	73	0.001	1.23(1.08–1.40)
Okumto 2004	55	0.01	1.62(1.28–2.03)	—	—	—
Our result from TCGA	62	0.003	1.78(1.35–2.34)	74	0.0008	1.76(1.25–2.48)
Ruan 2016 (Test)	61	0.004	1.69(1.30–2.21)	71	0.002	1.59(1.15–2.20)
Ruan 2016 (Validation)	58	0.007	1.64(1.29–2.09)	74	0.0008	1.60(1.16–2.19)
Tsai 1997 (May)	62	0.004	1.78(1.36–2.33)	—	—	—
Tsai 1997 (Aug)	61	0.005	1.78(1.38–2.30)	—	—	—
Turato 2014	—	—	—	51	0.06	1.74(1.31–2.31)
Wang 2016	62	0.004	1.68(1.31–2.16)	73	0.001	1.55(1.15–2.09)
Xiang 2011	—	—	—	73	0.001	1.56(1.15–2.12)

—, indicates there is no corresponding data presented.
P', the significance of heterogeneity, was artificially expressed as *p*'-value to distinguish from the significance of outcomes (p).
p'>0.1 was considered to be minor and acceptable heterogeneity.

Supplementary Table 4: Sensitivity tests for survival from COX multivariate analysis in HCC patients

Exclusion study	OS			DFS/MFS/PFS		
	I2 (%)	*p'*	HR(95%CI)	I^2 (%)	*p'*	HR(95%CI)
No exclusion	73	0.003	2.29(1.46–3.57)	81	0.005	1.62(0.66–3.98)
Chen 2013	70	0.009	2.02(1.34–3.04)	—	—	—
Gai 2014	78	0.001	2.67(1.38–5.14)	—	—	—
Ji 2015	68	0.01	1.96(1.28–3.00)	0	0.54	1.07(0.71–1.60)
Lin 2015	74	0.003	2.73(1.61–4.63)	88	0.003	1.99(0.39–10.11)
Okumto 2004	72	0.006	2.04(1.30–3.20)	—	—	—
Our result from TCGA	74	0.004	2.78(1.55–4.97)	—	—	—
Xiang 2011	—	—	—	88	0.004	2.21(0.57–8.50)

—, indicates there is no corresponding data presented.
p', the significance of heterogeneity, was artificially expressed as *p*'-value to distinguish from the significance of outcomes (p). *p* '> 0.1 was considered to be minor and acceptable heterogeneity.
